# Forecasting prognostic trajectories with mismatch negativity in early psychosis

**DOI:** 10.1017/S0033291721003068

**Published:** 2023-03

**Authors:** Minah Kim, Taekwan Kim, Wu Jeong Hwang, Silvia Kyungjin Lho, Sun-Young Moon, Tae Young Lee, Jun Soo Kwon

**Affiliations:** 1 Department of Neuropsychiatry, Seoul National University Hospital, Seoul, Republic of Korea; 2 Department of Psychiatry, Seoul National University College of Medicine, Seoul, Republic of Korea; 3 Department of Brain and Cognitive Sciences, Seoul National University College of Natural Sciences, Seoul, Republic of Korea; 4 Department of Neuropsychiatry, Pusan National University Yangsan Hospital, Yangsan, Republic of Korea; 5 Institute of Human Behavioral Medicine, SNU-MRC, Seoul, Republic of Korea

**Keywords:** Clinical high risk, early psychosis, event-related potential, first-episode psychosis, mismatch negativity, prognostic marker

## Abstract

**Background:**

Prognostic heterogeneity in early psychosis patients yields significant difficulties in determining the degree and duration of early intervention; this heterogeneity highlights the need for prognostic biomarkers. Although mismatch negativity (MMN) has been widely studied across early phases of psychotic disorders, its potential as a common prognostic biomarker in early periods, such as clinical high risk (CHR) for psychosis and first-episode psychosis (FEP), has not been fully studied.

**Methods:**

A total of 104 FEP patients, 102 CHR individuals, and 107 healthy controls (HCs) participated in baseline MMN recording. Clinical outcomes were assessed; 17 FEP patients were treatment resistant, 73 FEP patients were nonresistant, 56 CHR individuals were nonremitters (15 transitioned to a psychotic disorder), and 22 CHR subjects were remitters. Baseline MMN amplitudes were compared across clinical outcome groups and tested for utility prognostic biomarkers using binary logistic regression.

**Results:**

MMN amplitudes were greatest in HCs, intermediate in CHR subjects, and smallest in FEP patients. In the clinical outcome groups, MMN amplitudes were reduced from the baseline in both FEP and CHR patients with poor prognostic trajectories. Reduced baseline MMN amplitudes were a significant predictor of later treatment resistance in FEP patients [Exp(*β*) = 2.100, 95% confidence interval (CI) 1.104–3.993, *p* = 0.024] and nonremission in CHR individuals [Exp(*β*) = 1.898, 95% CI 1.065–3.374, *p* = 0.030].

**Conclusions:**

These findings suggest that MMN could be used as a common prognostic biomarker across early psychosis periods, which will aid clinical decisions for early intervention.

## Introduction

Early stages of psychotic disorder have been regarded as critical periods for early intervention to improve the clinical outcome of the disorder (Correll et al., 2018; Fusar-Poli, McGorry, & Kane, 2017; Lieberman et al., 2001). It has been well studied that a shorter duration of untreated psychosis (DUP) is related to a better prognosis of first-episode psychosis (FEP) patients (Marshall et al., 2005; Perkins, Gu, Boteva, & Lieberman, 2005). In addition, the concept of clinical high risk (CHR) for psychosis was established not only for early detection of FEP to shorten the DUP but also for delaying or preventing the onset of psychotic disorder (Miller et al., 2002; Yung et al., 2005). However, those patients in the early stages of psychotic disorder were proven to be heterogeneous in their prognostic trajectories. While approximately 40% of FEP patients show favorable outcomes, such as full remission or a partial response, the remaining patients display chronic, relapsing disease courses and are even treatment resistant (Birchwood, Todd, & Jackson, 1998; Lieberman, 1993). Similarly, three prognostic trajectories of positive, moderately impaired, and severely impaired outcomes were found among CHR individuals through group-based multitrajectory modeling (Allswede et al., 2020). Meta-analytic studies reported that 22% of CHR subjects transitioned to a psychotic disorder within 3 years and 65% of CHR individuals were nonremitters within 1.94 years (Fusar-Poli et al., 2013, 2020; Simon et al., 2013), suggesting that improving the general clinical outcome is as important as preventing the onset of psychotic disorder (Carrion et al., 2013; Lin et al., 2015; Schlosser et al., 2012).

Prognostic heterogeneity produces significant difficulties in clinical decision making, such as decisions concerning the early use of clozapine in FEP patients who are resistant to usual antipsychotic treatment. Intervention for CHR individuals also involves challenges, such as deciding who should receive rigorous or less intensive interventions, including the justification of the use of antipsychotic medication. By using biological predictors that will aid in forecasting prognostic trajectories of early psychosis patients, patients’ suffering during trial and error regarding treatment will be resolved, and a significant amount of time and resources will be saved. Previous studies reported that cortical gyrification (Palaniyappan et al., 2013), bilateral hippocampal increase (Lappin et al., 2014), corticostriatal functional connectivity (Oh, Kim, Kim, Lee, & Kwon, 2020; Sarpal et al., 2016), glutathione and glutamate levels (Dempster et al., 2020), and electrophysiological markers (Lho, Kim, Lee, Kwak, & Kwon, 2019; Mi et al., 2021; Renaldi et al., 2019) were associated with the treatment response of FEP patients. In CHR subjects, structural brain imaging (Cannon et al., 2015; de Wit et al., 2017; Ho et al., 2017; Koutsouleris et al., 2015; Koutsouleris, Upthegrove, & Wood, 2019; Reniers et al., 2017), neurochemical markers (Allen et al., 2015; Bossong et al., 2019; Egerton et al., 2014), and event-related potential (ERP) markers (Bodatsch et al., 2011; Hamilton et al., 2019; Kim, Lee, Lee, Kim, & Kwon, 2015; Kim, Lee, Yoon, Lee, & Kwon, 2018; Perez et al., 2014) were suggested as biological predictors of symptomatic and functional outcomes. However, clinically efficient biomarkers that forecast prognostic trajectories across the course of psychotic disorders, from CHR to FEP, have not yet been studied.

Mismatch negativity (MMN) is an ERP component that is elicited when repetitive standard stimuli are interrupted by infrequent deviant stimuli and is thus thought to be reflective of the automatic auditory change detection process (Naatanen & Escera, 2000). Because MMN generation is associated with neurotransmission at the N-methyl-D-aspartate (NMDA) receptor, MMN has been widely studied across the course of psychotic disorders to elucidate the pathophysiological mechanism of schizophrenia (Javitt & Freedman, 2015; Javitt, Steinschneider, Schroeder, & Arezzo, 1996; Uno & Coyle, 2019). Reduced duration deviant MMN (dMMN) amplitude has been consistently reported in schizophrenia and early psychosis patients, including FEP patients and CHR individuals, although the degree of dMMN impairment is less significant than in chronic schizophrenia patients (Erickson, Ruffle, & Gold, 2016; Haigh, Coffman, & Salisbury, 2017; Hamilton, Boos, & Mathalon, 2020; Kim, Cho, Yoon, Lee, & Kwon, 2017; Nagai et al., 2013; Tateno et al., 2021). Although little is known about dMMN as a prognostic predictor of FEP patients (Higgins, Lewandowski, Liukasemsarn, & Hall, 2021; Lho et al., 2020), previous studies, including our own, showed that dMMN was predictive of a transition to psychotic disorder, remission, and symptomatic and functional improvement in CHR individuals (Bodatsch et al., 2011; Fujioka et al., 2020; Kim et al., 2018; Perez et al., 2014). Therefore, dMMN has the potential to be a clinically efficient biomarker that forecasts prognostic trajectories across the course of psychotic disorder, which would aid in clinical decision making regarding interventions for patients with FEP and subjects at CHR for psychosis.

In the current study, we aimed to investigate whether dMMN amplitude could be a potential biomarker to predict poor prognostic outcomes in FEP and CHR individuals. We hypothesized that (1) dMMN is impaired in FEP and CHR participants compared to healthy controls (HCs); thus, dMMN may be a potential biomarker for prognosis prediction in early psychosis patients; (2) baseline dMMN is smaller in FEP patients who are treatment resistant than in patients who are not treatment resistant and is predictive of later treatment resistance; and (3) dMMN at baseline is reduced in CHR individuals who are not remitted or who transition to a psychotic disorder compared to individuals who are remitted or who have not transitioned after a minimum of 1 year from the baseline dMMN assessment, and dMMN is predictive of future nonremission from CHR status or transition to a psychotic disorder.

## Methods

### Participants

A total of 104 FEP patients, 102 individuals at CHR for psychosis, and 107 HCs participated in the baseline assessment, including dMMN recording, between August 2009 and June 2020. Among them, 25 FEP patients, 48 CHR subjects, and 47 HCs participated in our previous dMMN studies (Kim et al., 2017, 2018; Lho et al., 2019). FEP patients and CHR individuals were recruited from the Seoul Youth Clinic (SYC; www.youthclinic.org), a center for early detection and intervention of psychosis (Kwon, Byun, Lee, & An, 2012) and from an inpatient and outpatient clinic of the Department of Neuropsychiatry at the Seoul National University Hospital (SNUH). The definition of FEP was an individual aged 16–40 years who satisfied the diagnosis of schizophreniform disorder, schizophrenia or schizoaffective disorder when assessed using the Structured Clinical Interview for the Diagnostic and Statistical Manual of Mental Disorders, Fourth Edition, Axis I Disorders (SCID-I) and a duration of psychotic illness less than 2 years. Psychotic symptoms were assessed using the Positive and Negative Syndrome Scale (PANSS). To confirm the CHR status of the participants, the Structured Interview for Prodromal Symptoms (SIPS) (Miller et al., 2002) was used. Prodromal symptoms were assessed using the validated Korean version of the Scale of Prodromal Symptoms (SOPS) (Jung et al., 2010; Miller et al., 2003). In both the FEP and CHR groups, general functional status was defined using the modified Global Assessment of Functioning (mGAF) (Hall, 1995). HCs were recruited via internet advertisement and were screened using the SCID-I Nonpatient Edition (SCID-NP). Potential HC participants were excluded if they had any first- to third-degree biological relatives with a psychotic disorder. Common exclusion criteria included substance abuse or dependence (except nicotine), neurological disease or significant head trauma, medical illness that could be accompanied by psychiatric symptoms, sensory impairments, and intellectual disability [intelligence quotient (IQ) < 70].

Among the 104 FEP patients, 90 patients with FEP received usual treatment, including antipsychotic medication, for at least 18 months until June 2020. Treatment resistance was defined when a patient showed at least moderately severe psychotic symptoms despite taking a sufficient dose (⩾20 mg olanzapine equivalent dose per day) of more than two antipsychotics for at least 12 months or taking clozapine according to the minimum requirement criteria of the Treatment Response and Resistance in Psychosis (TRRIP) working group consensus guidelines (Conley & Buchanan, 1997; Gardner, Murphy, O'Donnell, Centorrino, & Baldessarini, 2010; Howes et al., 2017). Certified psychiatrists who were blinded to the dMMN amplitudes thoroughly reviewed medical records to assess the symptom severity, medication status, and treatment adherence information provided by patients themselves and their caregivers at each visit to clinic. As a result, FEP patients were divided into treatment-resistant (FEP-TR, *n* = 17) and nonresistant (FEP-nonTR, *n* = 73) groups. The means and standard deviations of follow-up duration were 39.4 ± 32.7 months in the FEP-TR group and 46.1 ± 34.2 months in the FEP-nonTR group. Among the 102 CHR subjects, 78 CHR individuals participated in annual follow-up clinical assessment at least once until June 2020. A remitter was defined as a CHR individual who scored <3 on the SOPS positive symptoms subscale and 65⩾ on the mGAF measured at the last clinical assessment to reflect both symptomatic and functional recovery (Bossong et al., 2019; Fujioka et al., 2020; Hamilton et al., 2019; Kim et al., 2018). According to the remission criteria, certified psychiatrists who were blinded to dMMN amplitude determined 22 CHR remitters and 56 nonremitters, including 15 subjects who ended the follow-up assessment with a transition to a psychotic disorder (12 schizophrenia and three schizoaffective disorder) according to SIPS criteria. The means and standard deviations of follow-up duration were 46.0 ± 36.5 months for CHR remitters and 30.1 ± 27.9 months for CHR nonremitters. Sixty-three CHR individuals did not transition to psychotic disorder (online Fig. S1 in the Supplementary Material).

Written informed consent was obtained from all participants after they were given a thorough explanation of the study procedure (IRB no. H-1110-009-380). For minors, informed consent was obtained from both the participants themselves and their parents. This study was conducted in accordance with the Declaration of Helsinki and was approved by the Institutional Review Board of SNUH (IRB no. H-2008-195-1154).

### MMN acquisition

Continuous electroencephalographic (EEG) recording was conducted using a Neuroscan 128 Channel SynAmps system equipped with a 128-channel Quick-Cap based on the modified 10–20 international system (Compumedics, Charlotte, NC, USA) while participants were performing a passive auditory oddball task. The EEG data were digitized at a 1000 Hz sampling rate, and an online bandpass filter between 0.05 and 100 Hz was used. The reference electrodes were placed on both mastoids. The vertical and horizontal electrooculograms were recorded using electrodes below and on the outer canthus of the left eye to monitor eye movement artifacts. The resistance of all electrode sites was less than 5 kΩ. During the passive auditory oddball task performance, participants were instructed to ignore the auditory sound and concentrate on a ‘Where's Waldo?’ picture book. A pseudorandom series of 1000 Hz (80 dB, 10 ms rise/fall) auditory stimuli were binaurally presented using a STIM2 sound generator (Compumedics). With an intertrial interval of 600 ms, the duration of deviant stimuli was 100 ms (18.2%, 218/1200), and the duration of standard stimuli was 50 ms (81.8%, 982/1200).

### Data preprocessing

Curry version 7 software (Compumedics) was used to preprocess the ERP data. After replacing bad channels using the linear interpolation of the adjacent channels (up to 7%), eye movement artifact reduction was performed according to the validated ocular artifact reduction algorithm (Semlitsch, Anderer, Schuster, & Presslich, 1986). EEG recordings were rereferenced to common average reference data, and a 0.1–30 Hz bandpass filter was applied. Continuous EEG data were epoched to a 100 ms prestimulus interval and a 300 ms poststimulus interval, and the averaged prestimulus interval voltage was used in baseline correction. Automatic artifact rejection was performed by removing the epochs containing EEG amplitudes that exceeded ± 75 *μ*V. The means and standard deviations of the numbers of remaining epochs for deviant stimuli were not significantly different across all groups (Table 1). dMMN was calculated by subtracting the ERPs elicited by the standard stimuli from those elicited by deviant stimuli. The most negative deflection between 130 and 250 ms poststimulus onset at the FCz electrode site, where dMMN had the maximal amplitude (Garrido, Kilner, Stephan, & Friston, 2009), was detected as the peak dMMN amplitudes and latencies.

**Table 1.** Demographic, clinical, and duration deviant mismatch negativity (dMMN) characteristics of the participants at baseline

	Group, no (%)			Statistical analysis^1^		FEP clinical outcome group, no (%)		Statistical analysis^2^		CHR clinical outcome group, no (%)		Statistical analysis^3^	
Characteristics	FEP (*n* = 104)	CHR (*n* = 102)	HC (*n* = 107)	*F* or *T* or χ^2^	*p*	FEP-TR (*n* = 17)	FEP-nonTR (*n* = 73)	χ^2^ or *Z*	*p*	CHR remitters (*n* = 22)	CHR nonremitters (*n* = 56)	χ^2^ or *T*	*p*
Sex (male/female)	41/63	75/27	74/33	29.984	<0.001**	8/9	29/44	0.306	0.580	16/6	40/16	0.013	0.909
Handedness (right/left)	97/7	97/5	101/6	0.324	0.850	15/2	69/4	0.875	0.349	22/0	52/4	1.656	0.198
Age (years)	23.4 ± 4.8	20.3 ± 3.7	22.8 ± 4.3	15.988	<0.001**	23.2 ± 4.5	23.6 ± 5.2	−0.201	0.840	19.9 ± 3.1	20.5 ± 3.8	−0.643	0.522
IQ	100.2 ± 14.6	105.6 ± 13.2	114.6 ± 12.8	30.292	<0.001**	96.8 ± 18.3	101.6 ± 13.5	−1.516	0.129	105.8 ± 14.6	104.3 ± 12.0	0.474	0.637
Education (years)	14.0 ± 2.6	12.5 ± 1.8	13.9 ± 2.2	16.726	<0.001**	14.2 ± 3.9	13.9 ± 2.3	−0.404	0.686	12.5 ± 1.4	12.6 ± 1.8	−0.244	0.808
DOI (months)	8.2 ± 6.3	–	–	–	–	10.6 ± 5.9	6.9 ± 5.1	−2.377	0.017*	–	–	–	–
DUP (months)	4.6 ± 4.7	–	–	–	–	5.7 ± 5.4	4.0 ± 4.2	−0.745	0.457	–	–	–	–
DUPP (months)	–	17.8 ± 17.8	–	–	–	–	–	–	–	19.5 ± 22.9	17.4 ± 16.4	0.455	0.651
PANSS													
Positive symptoms	15.2 ± 5.5	–	–	–	–	16.9 ± 5.0	14.5 ± 5.4	−1.751	0.080	–	–	–	–
Negative symptoms	16.1 ± 6.1	–	–	–	–	16.5 ± 6.3	16.1 ± 5.9	−0.341	0.733	–	–	–	–
General symptoms	31.8 ± 9.1	–	–	–	–	29.9 ± 9.2	31.7 ± 8.5	−0.671	0.502	–	–	–	–
SOPS													
Positive symptoms	–	9.8 ± 3.6	–	–	–	–	–	–	–	9.3 ± 3.6	9.8 ± 3.8	−0.476	0.636
Negative symptoms	–	13.8 ± 6.7	–	–	–	–	–	–	–	13.3 ± 7.1	14.3 ± 6.5	−0.612	0.542
Disorganization	–	4.3 ± 2.9	–	–	–	–	–	–	–	4.3 ± 2.6	4.6 ± 3.1	−0.390	0.698
General symptoms	–	6.9 ± 3.9	–	–	–	–	–	–	–	7.0 ± 3.8	7.3 ± 4.1	−0.281	0.779
mGAF	50.8 ± 13.1	53.2 ± 9.7	–	−1.541	0.125	46.8 ± 16.0	51.7 ± 12.7	−1.181	0.237	50.3 ± 9.5	52.0 ± 9.0	−0.769	0.444
Prescribed medication^4^													
Antipsychotics	95 (91.3)	14 (13.7)	–	124.525	<0.001**	17 (100.0)	65 (89.0)	2.045	0.153	3 (13.6)	7 (12.5)	0.018	0.893
Antidepressants	12 (9.6)	25 (24.5)	–	5.880	0.015*	3 (17.6)	8 (11.0)	0.575	0.448	4 (18.2)	17 (30.4)	1.190	0.275
Mood stabilizers	10 (9.6)	3 (2.9)	–	3.880	0.049*	2 (11.8)	7 (9.6)	1.540	0.215	1 (4.5)	1 (1.8)	0.482	0.488
Anxiolytics	61 (58.7)	26 (25.5)	–	23.214	<0.001**	11 (64.7)	41 (56.2)	0.412	0.521	5 (22.7)	17 (30.4)	0.454	0.500
dMMN peak amplitudes (*μ*V)													
FCz electrode site	−1.5 ± 1.1	−2.0 ± 1.0	−2.5 ± 1.3	19.915	<0.001**	−1.0 ± 1.0	−1.7 ± 1.2	−2.093	0.036*	−2.3 ± 1.2	−1.8 ± 0.8	−2.136	0.036*
dMMN peak latencies (ms)													
FCz electrode site	185.0 ± 33.6	192.1 ± 30.0	190.7 ± 29.5	1.009	0.366	178.0 ± 36.7	178.4 ± 28.7	−0.144	0.885	185.6 ± 18.8	191.5 ± 34.0	−0.771	0.443
Number of remaining epochs for deviant stimuli	191.3 ± 27.4	192.6 ± 25.5	189.3 ± 27.4	0.395	0.674	189.4 ± 29.1	191.6 ± 27.1	−0.160	0.873	198.1 ± 18.1	190.6 ± 28.2	1.148	0.255

FEP, first-episode psychosis; CHR, clinical high risk; HC, healthy control; FEP-TR, FEP patients who were treatment resistant; FEP-nonTR, FEP patients who were not treatment resistant; IQ, intelligence quotient; DOI, duration of illness; DUP, duration of untreated psychosis; DUPP, duration of untreated prodromal psychosis; PANSS, Positive and Negative Syndrome Scale; SOPS, Scale of Prodromal Symptoms; mGAF, modified Global Assessment of Functioning.

Data are indicated as the mean ± standard deviation.

*Statistical significance at *p* < 0.05.

**Statistical significance at *p* < 0.005.

1 Analysis of variance, independent *t* test or Welch's *t* test if the variances were not equal; χ^2^ analysis or Fisher's exact test for categorical data.

2 Mann–Whitney U test, χ^2^ analysis or Fisher's exact test for categorical data.

3 Independent *t* test or Welch's *t* test if the variances were not equal; χ^2^ analysis or Fisher's exact test for categorical data.

4 Number (percentage) of participants who were prescribed each medication at the time of dMMN measurement.

### Statistical analysis

The demographic, clinical, and dMMN characteristics were compared using analysis of variance (ANOVA) or independent *t* test across the groups for continuous variables. A Bonferroni test was used for *post hoc* analysis. Welch's *t* test was performed if the variances were not equal, and Mann–Whitney *U* tests were used if the normality assumption was not satisfied. Chi-square tests were used for categorical variables. Group comparisons of MMN amplitudes and latencies across the FEP patients, CHR subjects, and HCs were performed using univariate analysis of covariance (ANCOVA) with age as a covariate. A *post hoc* simple contrast test was used to reveal specific group differences. Binary logistic regression analyses with the backward selection method were used to investigate whether baseline dMMN amplitudes were predictive of treatment resistance in FEP patients and predictive of nonremission or transition to psychotic disorder in CHR individuals. Common independent variables were the dMMN amplitude at FCz electrode site, sex, handedness, age, IQ, and education years in both FEP and CHR groups. Variables with significant group differences at baseline were selected as additional independent variables, which included the duration of illness (DOI) for FEP treatment resistance prediction and the SOPS positive subscale score at baseline for CHR transition prediction. Statistical analyses were performed using SPSS v.25.0 (IBM, Armonk, NY), and statistical significance was set at *p* < 0.05.

## Results

### Participant characteristics

Table 1 summarizes the demographic and clinical characteristics of the participants at baseline. Detailed information including follow-up characteristics of the clinical outcome groups is presented in online Supplementary Table S1 (FEP-TR *v.* FEP-nonTR) and online Supplementary Table S2 (CHR remitters *v.* CHR nonremitters and transitioned CHR *v.* nontransitioned CHR) in the Supplementary Material. There were more females than males in the FEP group than in the CHR and HC groups (χ^2^ = 29.984, *p* < 0.001). Individuals at CHR for psychosis were significantly younger and less educated than FEP patients (age, *p* < 0.001; education years, *p* < 0.001) and HCs (age, *p* < 0.001; education years, *p* < 0.001). IQ was highest in HCs (HC *v.* FEP, *p* < 0.001; HC *v.* CHR, *p* < 0.001) and lowest in FEP patients (FEP *v.* CHR, *p* = 0.014). In the clinical outcome group comparison, FEP-TR patients had a longer DOI than FEP-nonTR patients (*Z* = −2.377, *p* = 0.017), and transitioned CHR subjects had higher scores on the SOPS positive symptoms subscale (*Z* = −2.018, *p* = 0.044) than nontransitioned CHR individuals at baseline. Other baseline demographic and clinical characteristics, as well as follow-up duration, were not significantly different across the clinical outcome groups. Online Tables S3 and S4 in the Supplementary Material show no significant difference in baseline participant characteristics between the FEP patients who were assessed as treatment resistant and those who were not, as well as between CHR subjects who participated in the follow-up assessment and those who did not.

### MMN characteristics

**Fig. 1.**
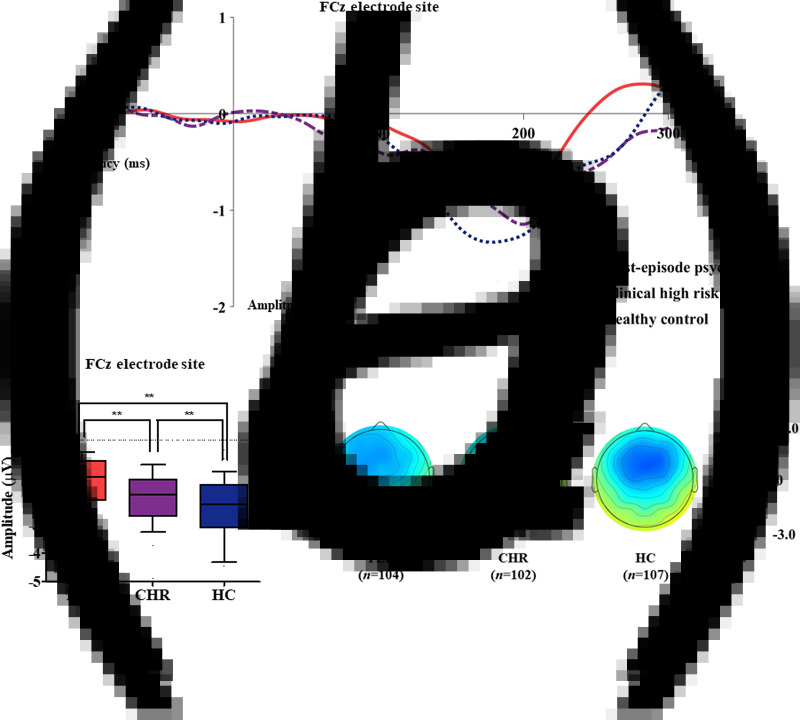
(*a*) Grand-averaged duration deviant mismatch negativity (dMMN) waveforms across the patients with first-episode psychosis (FEP), subjects at clinical high risk (CHR) for psychosis, and healthy controls (HCs) at the FCz electrode site. (*b*) dMMN amplitude across the groups at the FCz electrode site. Horizontal lines in groups indicate means, and vertical lines indicate 95% confidence intervals. *Indicates statistical significance at *p* < 0.05. **Indicates statistical significance at *p* < 0.005. (*c*) Two-dimensional topographic maps of dMMN in FEP patients, CHR individuals, and HCs. The colored bar with numbers indicates the dMMN amplitude (*μ*V).

**Fig. 2.**
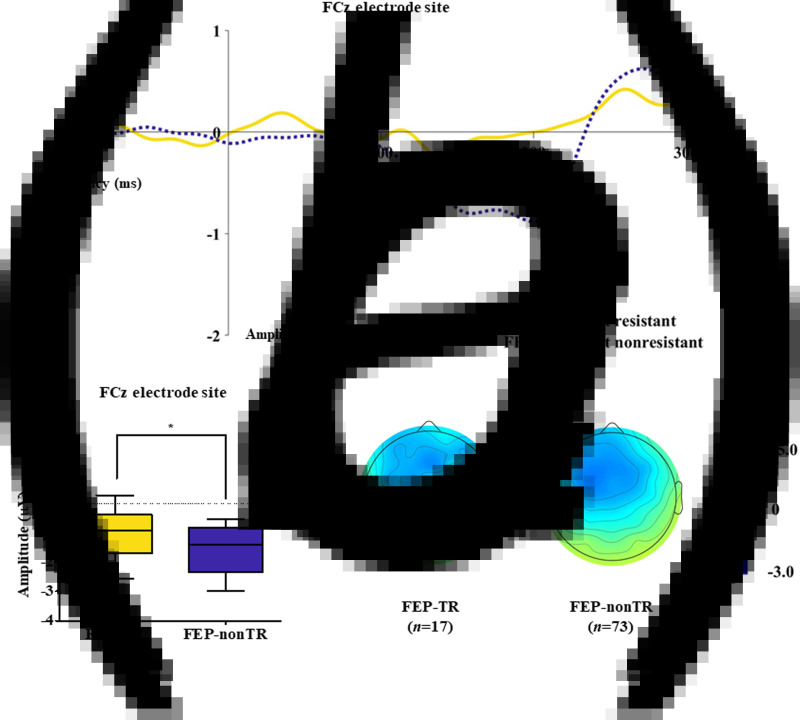
(*a*) Grand-averaged duration deviant mismatch negativity (dMMN) waveforms across the patients with first-episode psychosis (FEP) who were treatment resistant (FEP-TR) and those who were not (FEP-nonTR) at the FCz electrode site. (*b*) dMMN amplitude across the clinical outcome groups at the FCz electrode site. Horizontal lines in groups indicate means, and vertical lines indicate 95% confidence intervals. *Indicates statistical significance at *p* < 0.05. (*c*) Two-dimensional topographic maps of dMMN in FEP-TR and FEP-nonTR patients. The colored bar with numbers indicates the amplitude of the dMMN (*μ*V).

**Fig. 3.**
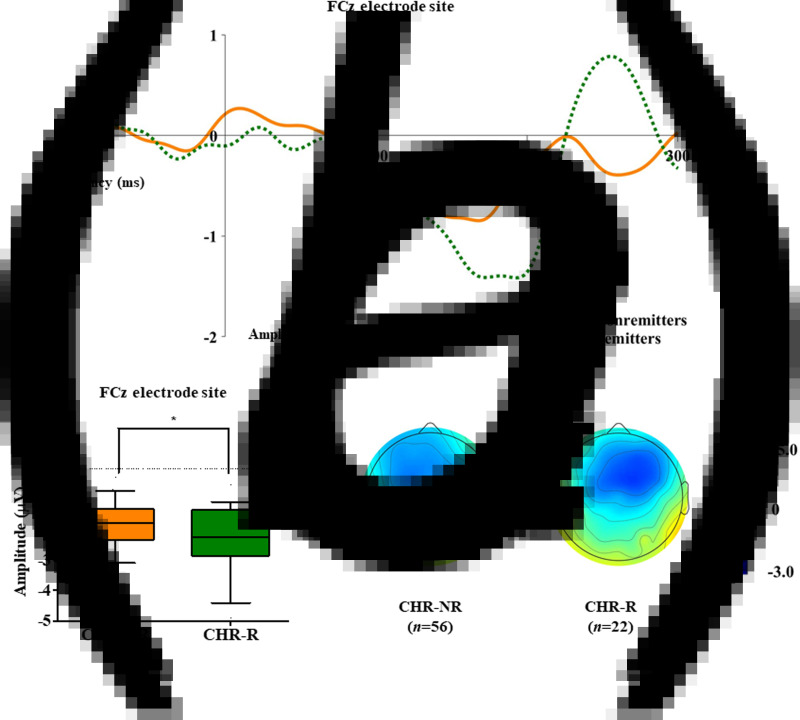
(*a*) Grand-averaged duration deviant mismatch negativity (dMMN) waveforms across the individuals at clinical high risk (CHR) for psychosis who were remitted and those who were not at the FCz electrode site. (*b*) dMMN amplitude across the clinical outcome groups at the FCz electrode site. Horizontal lines in groups indicate means, and vertical lines indicate 95% confidence intervals. *Indicates statistical significance at *p* < 0.05. (*c*) Two-dimensional topographic maps of dMMN in CHR remitters and CHR nonremitters. The colored bar with numbers indicates the amplitude of the dMMN (*μ*V).

Table 1 presents group comparison results of baseline dMMN peak amplitudes and latencies at the FCz electrode site. ANCOVA with age as a covariate revealed a significant group difference in dMMN amplitude at the FCz electrode site (*F* = 19.915, *p* < 0.001) across the FEP, CHR, and HC groups. A *post hoc* simple contrast test showed that dMMN amplitudes were greatest in HCs, intermediate in CHR subjects, and smallest in FEP patients (FEP *v.* HC, *p* < 0.001; CHR *v.* HC, *p* = 0.002; FEP *v.* CHR, *p* = 0.004; Fig. 1). There was no significant group effect of dMMN peak latency. According to the clinical outcome groups, the FEP-TR group showed smaller dMMN amplitudes than the FEP-nonTR group (*Z* = −2.093, *p* = 0.036, Cohen's *d* = 0.634; Fig. 2), and dMMN amplitudes of CHR remitters were greater than those of CHR nonremitters (*t* = −2.136, *p* = 0.036, Cohen's *d* = 0.544; Fig. 3). In addition, ANCOVA with age as a covariate showed that dMMN amplitudes were significantly different across FEP patients, CHR nonremitters, CHR remitters, and HCs (*F* = 14.435, *p* < 0.001). A *post hoc* simple contrast test revealed that dMMN amplitudes were similarly impaired in FEP patients and CHR nonremitters compared to CHR remitters and HCs (FEP *v.* CHR nonremitters, *p* = 0.164; FEP *v.* CHR remitters, *p* = 0.004; CHR remitters *v.* HC, *p* = 0.441; online Fig. S2 in the Supplementary Material). Group comparison results across the FEP-TR group, FEP-nonTR group, CHR nonremitters, CHR remitters, and HCs are provided in the online Supplementary Material (Fig. S3). No significant group difference in dMMN amplitude was found between CHR subjects who transitioned to a psychotic disorder and those who did not (online Table S5 in the Supplementary Material). Online Tables S6 and S7 in the Supplementary Material show that there was no significant difference in dMMN characteristics between FEP patients who were assessed as treatment resistant and those who were not or between CHR subjects who participated in the follow-up assessment and those who did not.

### Predicting prognostic trajectories using MMN

Table 2 presents the results of binary logistic regression analysis with the backward selection method. Treatment resistance in FEP was predicted by the dMMN amplitude [Exp(*β*) = 2.100, 95% confidence interval (95% CI) 1.104–3.993, *p* = 0.024] and DOI [Exp(*β*) = 1.138, 95% CI 1.031–1.256, *p* = 0.010]. Nonremission in CHR patients was predicted by the dMMN amplitude [Exp(*β*) = 1.895, 95% CI 1.065–3.374, *p* = 0.030]. The binary logistic regression model for predicting the transition to a psychotic disorder in CHR subjects did not include the dMMN amplitude but only included the SOPS positive subscale score at baseline [Exp(*β*) = 1.178, 95% CI 1.005–1.380, *p* = 0.043].

**Table 2.** Significant predictors of treatment resistance in first-episode psychosis (FEP) patients and nonremission or transition to psychotic disorder in subjects at clinical high risk (CHR) for psychosis in binary logistic regression analysis with the backward selection method

Clinical outcome	Significant predictors	*R*^2^	Exp (*B*)	*p*	95% CI	
					Lower	Upper
Treatment resistance in FEP	dMMN peak amplitude at FCz	0.206	2.100	0.024*	1.104	3.993
	duration of illness		1.138	0.010*	1.031	1.256
Nonremission in CHR	dMMN peak amplitude at FCz	0.142	1.898	0.030*	1.065	3.374
Transition in CHR	SOPS positive symptoms at baseline	0.088	1.178	0.043*	1.005	1.380

CI, confidence interval; dMMN, duration deviant mismatch negativity; SOPS, Scale of Prodromal Symptoms.

*Statistical significance, *p* < 0.05.

## Discussion

Diagnosis based on the symptomatic phenotype produces significant prognostic heterogeneity that interferes with the goal of early intervention in early psychosis; thus, investigation of biomarkers that are predictive of prognostic trajectories of early psychosis patients is warranted (Allswede et al., 2020; Birchwood et al., 1998; Clementz et al., 2016). In this longitudinal study, we aimed to confirm the usefulness of dMMN as a common prognostic biomarker across the early psychosis periods, from CHR to FEP. In line with many previous studies (Erickson et al., 2016; Higgins et al., 2021; Tateno et al., 2021), dMMN amplitudes at the FCz electrode site were impaired in both FEP and CHR patients; thus, these amplitudes were used for further analysis for prognosis prediction. From the baseline assessment, dMMN amplitude was smaller in FEP-TR patients than in FEP-nonTR patients and was predictive of later treatment resistance. In addition, the dMMN amplitude was reduced in CHR nonremitters compared to CHR remitters at baseline and was associated with nonremission from a CHR status. These findings not only support the need for biomarkers predictive of prognostic trajectories but also highlight the usefulness of dMMN as a common prognostic biomarker across the early psychosis periods.

In the current study, impaired dMMN amplitude was observed from the baseline in FEP patients with poor prognosis (i.e. FEP-TR) and was a significant predictor of later treatment resistance. Considering that MMN generation is closely related to NMDA receptor-mediated glutamatergic activity (Javitt et al., 1996; Uno & Coyle, 2019), the current study result is in line with previous studies that reported an abnormal NMDA-glutamate system and its association with poor treatment response in FEP patients (Dempster et al., 2020; Mouchlianitis et al., 2016). The present study suggests that FEP patients with reduced dMMN amplitude may benefit from the early use of clozapine or adjuvant use of drugs targeting the NMDA-glutamate system (Rapado-Castro et al., 2017; Swerdlow et al., 2016; Zheng et al., 2018). In addition, recent studies have shown that treatment resistance to antipsychotics is evident upon illness onset in most FEP-TR patients (Demjaha et al., 2017; Lally et al., 2016), suggesting that FEP treatment resistance can be forecasted by biomarkers showing abnormalities from the early psychosis period, such as MMN.

We found that CHR nonremitters presented a reduced dMMN amplitude, which was comparable to that of FEP patients and was smaller than that of HCs, whereas CHR remitters showed a similar magnitude of dMMN amplitude as that of HCs, which was larger than that of FEP patients. This finding supports the biological heterogeneity of CHR individuals, which may be a cause of the smaller effect size of MMN impairment found in the entire CHR group compared to FEP and schizophrenia patients (Erickson et al., 2016; Kim et al., 2017). Furthermore, we replicated our previous report with extended cohort data and showed that a relatively preserved dMMN amplitude to a level of HCs at baseline was associated with a better clinical outcome, such as remission (Kim et al., 2018); a similar result was provided by a recent study by Fujioka et al. (2020). However, our analysis did not replicate previous study results that reported that impaired MMN was predictive of later transition to psychotic disorder (Bodatsch et al., 2011; Perez et al., 2014). The relatively small number of transitioned CHR subjects and the prescription of adequate medication, which aims to reduce the transition rate, may be the cause of the insignificant results of the current study. Therefore, future large-scale multisite longitudinal studies controlling for medication are warranted to test whether MMN could predict the prognosis of CHR individuals, including remission, nonremission, and transition to psychotic disorder, as in the recent study by Hamilton et al. (2019) of auditory P300.

Although treatment guidelines for schizophrenia recommend the use of clozapine in patients who are resistant to usual antipsychotic treatment (Addington, Addington, Abidi, Raedler, & Remington, 2017), it is difficult to know which FEP patients will be treatment resistant without spending a significant amount of time and resources and without extending patients’ suffering during trial and error. Moreover, in subjects at CHR for psychosis, although deciding the timing and intensity of intervention is more critical due to the nonspecific, heterogeneous, and changing nature of at-risk states, it is not easy to determine when to provide an intervention, which patients should receive an intervention, and the most suitable type of intervention to use due to the uncertain, diverse clinical outcomes of subjects at CHR for psychosis (Fusar-Poli et al., 2020). In addition, existing studies proposing CHR risk calculators based on clinical and neurocognitive features suggest the importance of the incorporation of biomarkers, such as ERP components reflective of early psychosis states, to enhance model performance (Cannon et al., 2016; Oribe et al., 2020; Park et al., 2021; Schmidt et al., 2017). Given that early psychosis periods exhibit a continuum of prodrome to FEP states, which share the common aspects of improved clinical outcomes by early interventions (Correll et al., 2018; Fusar-Poli et al., 2017; Lieberman et al., 2001), common prognostic biomarkers that forecast prognosis across the early psychosis periods would provide valuable information for early intervention. In this regard, this study first suggested that MMN may serve as a common prognostic biomarker that should be included in future prediction model development to forecast clinical outcomes of early psychosis patients from CHR to FEP states.

This study has several limitations. First, medication use at the time of dMMN recording and the time to clinical outcome varied among FEP patients and CHR individuals; however, medication use at baseline and follow-up duration between clinical outcome groups were not different. Second, treatment resistance and nonremission were determined at a single last assessment that could not reflect changes outside the assessment points; thus, the findings should be interpreted with caution and potential biases should be considered. For example, a CHR individual who was classified as a remitter at the last follow-up assessment could subsequently become a nonremitter or transition to a psychotic disorder. Third, we used dMMN exclusively, unlike previous studies that used frequency-deviant MMN or double-deviant MMN (Bodatsch et al., 2011; Perez et al., 2014), which may be one of the causes of the negative results in the exploratory analysis for predicting the transition to psychotic disorder. Fourth, our sample size and the number of FEP-TR and transitioned CHR patients were small compared to other multisite studies, which may have diluted the effect of MMN on prognosis prediction. Considering that our sample was obtained from a single center (i.e. SYC), the sample size was relatively large, and our results are free from the issue of multicenter data stability. Nevertheless, large-scale multisite longitudinal studies with various MMN paradigms should be conducted to confirm the results of the current study.

To our knowledge, the present study is the first to investigate whether dMMN could serve as a common prognostic biomarker in early psychosis patients across the CHR to FEP. We observed that a reduced dMMN amplitude was already present at the baseline in poor clinical outcome groups and was predictive of treatment resistance in FEP patients and nonremission in subjects at CHR for psychosis. The present study suggests that reduced dMMN amplitude may support the clinical decision of early clozapine use for expected FEP-TR and the provision of rigorous treatment for potential CHR nonremission cases. In conclusion, the current study results provide information regarding dMMN as a common prognostic biomarker that could be added to existing prediction models based on clinical and neurocognitive features across early psychosis periods, which will aid in clinical decision-making for early interventions.

## Supplementary material

For supplementary material accompanying this paper visit https://doi.org/10.1017/S0033291721003068. click here to view supplementary material

## References

[ref1] Addington, J., Addington, D., Abidi, S., Raedler, T., & Remington, G. (2017). Canadian treatment guidelines for individuals at clinical high risk of psychosis. The Canadian Journal of Psychiatry, 62(9), 656–661. doi:10.1177/0706743717719895. 28730848 PMC5593244

[ref2] Allen, P., Chaddock, C. A., Egerton, A., Howes, O. D., Barker, G., Bonoldi, I., … McGuire, P. (2015). Functional outcome in people at high risk for psychosis predicted by thalamic glutamate levels and prefronto-striatal activation. Schizophrenia Bulletin, 41(2), 429–439. doi:10.1093/schbul/sbu115. 25123110 PMC4332951

[ref3] Allswede, D. M., Addington, J., Bearden, C. E., Cadenhead, K. S., Cornblatt, B. A., Mathalon, D. H., … Cannon, T. D. (2020). Characterizing covariant trajectories of individuals at clinical high risk for psychosis across symptomatic and functional domains. American Journal of Psychiatry, 177(2), 164–171. doi:10.1176/appi.ajp.2019.18111290. 31509005 PMC7002249

[ref4] Birchwood, M., Todd, P., & Jackson, C. (1998). Early intervention in psychosis. The critical period hypothesis. The British Journal of Psychiatry, 172(33), 53–59. doi:10.1192/S0007125000297663. 9764127

[ref5] Bodatsch, M., Ruhrmann, S., Wagner, M., Muller, R., Schultze-Lutter, F., Frommann, I., … Brockhaus-Dumke, A. (2011). Prediction of psychosis by mismatch negativity. Biological Psychiatry, 69(10), 959–966. doi:10.1016/j.biopsych.2010.09.057. 21167475

[ref6] Bossong, M. G., Antoniades, M., Azis, M., Samson, C., Quinn, B., Bonoldi, I., … McGuire, P. (2019). Association of hippocampal glutamate levels with adverse outcomes in individuals at clinical high risk for psychosis. JAMA Psychiatry, 76(2), 199–207. doi:10.1001/jamapsychiatry.2018.3252. 30427993 PMC6440239

[ref7] Cannon, T. D., Chung, Y., He, G., Sun, D., Jacobson, A., van Erp, T. G., … North American Prodrome Longitudinal Study Consortium. (2015). Progressive reduction in cortical thickness as psychosis develops: A multisite longitudinal neuroimaging study of youth at elevated clinical risk. Biological Psychiatry, 77(2), 147–157. doi:10.1016/j.biopsych.2014.05.023. 25034946 PMC4264996

[ref8] Cannon, T. D., Yu, C., Addington, J., Bearden, C. E., Cadenhead, K. S., Cornblatt, B. A., … Kattan, M. W. (2016). An individualized risk calculator for research in prodromal psychosis. American Journal of Psychiatry, 173(10), 980–988. doi:10.1176/appi.ajp.2016.15070890. 27363508 PMC5048498

[ref9] Carrion, R. E., McLaughlin, D., Goldberg, T. E., Auther, A. M., Olsen, R. H., Olvet, D. M., … Cornblatt, B. A. (2013). Prediction of functional outcome in individuals at clinical high risk for psychosis. JAMA Psychiatry, 70(11), 1133–1142. doi:10.1001/jamapsychiatry.2013.1909. 24006090 PMC4469070

[ref10] Clementz, B. A., Sweeney, J. A., Hamm, J. P., Ivleva, E. I., Ethridge, L. E., Pearlson, G. D., … Tamminga, C. A. (2016). Identification of distinct psychosis biotypes using brain-based biomarkers. American Journal of Psychiatry, 173(4), 373–384. doi:10.1176/appi.ajp.2015.14091200. 26651391 PMC5314432

[ref11] Conley, R. R., & Buchanan, R. W. (1997). Evaluation of treatment-resistant schizophrenia. Schizophrenia Bulletin, 23(4), 663–674. doi:10.1093/schbul/23.4.663. 9366002

[ref12] Correll, C. U., Galling, B., Pawar, A., Krivko, A., Bonetto, C., Ruggeri, M., … Kane, J. M. (2018). Comparison of early intervention services vs treatment as usual for early-phase psychosis: A systematic review, meta-analysis, and meta-regression. JAMA Psychiatry, 75(6), 555–565. doi:10.1001/jamapsychiatry.2018.0623. 29800949 PMC6137532

[ref13] Demjaha, A., Lappin, J. M., Stahl, D., Patel, M. X., MacCabe, J. H., Howes, O. D., … Murray, R. M. (2017). Antipsychotic treatment resistance in first-episode psychosis: Prevalence, subtypes and predictors. Psychological Medicine, 47(11), 1981–1989. doi:10.1017/S0033291717000435. 28395674

[ref14] Dempster, K., Jeon, P., MacKinley, M., Williamson, P., Theberge, J., & Palaniyappan, L. (2020). Early treatment response in first episode psychosis: A 7-T magnetic resonance spectroscopic study of glutathione and glutamate. Molecular Psychiatry, 25(8), 1640–1650. doi:10.1038/s41380-020-0704-x. 32205866 PMC7387300

[ref15] de Wit, S., Ziermans, T. B., Nieuwenhuis, M., Schothorst, P. F., van Engeland, H., Kahn, R. S., … Schnack, H. G. (2017). Individual prediction of long-term outcome in adolescents at ultra-high risk for psychosis: Applying machine learning techniques to brain imaging data. Human Brain Mapping, 38(2), 704–714. doi:10.1002/hbm.23410. 27699911 PMC6866746

[ref16] Egerton, A., Stone, J. M., Chaddock, C. A., Barker, G. J., Bonoldi, I., Howard, R. M., … McGuire, P. K. (2014). Relationship between brain glutamate levels and clinical outcome in individuals at ultra high risk of psychosis. Neuropsychopharmacology, 39(12), 2891–2899. doi:10.1038/npp.2014.143. 24917199 PMC4180719

[ref17] Erickson, M. A., Ruffle, A., & Gold, J. M. (2016). A meta-analysis of mismatch negativity in schizophrenia: From clinical risk to disease specificity and progression. Biological Psychiatry, 79(12), 980–987. doi:10.1016/j.biopsych.2015.08.025. 26444073 PMC4775447

[ref18] Fujioka, M., Kirihara, K., Koshiyama, D., Tada, M., Nagai, T., Usui, K., … Kasai, K. (2020). Mismatch negativity predicts remission and neurocognitive function in individuals at ultra-high risk for psychosis. Frontiers in Psychiatry, 11, 770. doi:10.3389/fpsyt.2020.00770. 32848939 PMC7416637

[ref19] Fusar-Poli, P., Bechdolf, A., Taylor, M. J., Bonoldi, I., Carpenter, W. T., Yung, A. R., & McGuire, P. (2013). At risk for schizophrenic or affective psychoses? A meta-analysis of DSM/ICD diagnostic outcomes in individuals at high clinical risk. Schizophrenia Bulletin, 39(4), 923–932. doi:10.1093/schbul/sbs060. 22589370 PMC3686446

[ref20] Fusar-Poli, P., de Pablo, G. S., Correll, C. U., Meyer-Lindenberg, A., Millan, M. J., Borgwardt, S., … Arango, C. (2020). Prevention of psychosis: Advances in detection, prognosis, and intervention. JAMA Psychiatry, 77(7), 755–765. doi:10.1001/jamapsychiatry.2019.4779. 32159746

[ref21] Fusar-Poli, P., McGorry, P. D., & Kane, J. M. (2017). Improving outcomes of first-episode psychosis: An overview. World Psychiatry, 16(3), 251–265. doi:10.1002/wps.20446. 28941089 PMC5608829

[ref22] Gardner, D. M., Murphy, A. L., O'Donnell, H., Centorrino, F., & Baldessarini, R. J. (2010). International consensus study of antipsychotic dosing. American Journal of Psychiatry, 167(6), 686–693. doi:10.1176/appi.ajp.2009.09060802. 20360319

[ref23] Garrido, M. I., Kilner, J. M., Stephan, K. E., & Friston, K. J. (2009). The mismatch negativity: A review of underlying mechanisms. Clinical Neurophysiology, 120(3), 453–463. doi:10.1016/j.clinph.2008.11.029. 19181570 PMC2671031

[ref24] Haigh, S. M., Coffman, B. A., & Salisbury, D. F. (2017). Mismatch negativity in first-episode schizophrenia: A meta-analysis. Clinical EEG and Neuroscience, 48(1), 3–10. doi:10.1177/1550059416645980. 27170669 PMC5768309

[ref25] Hall, R. C. (1995). Global assessment of functioning. A modified scale. Psychosomatics, 36(3), 267–275. doi:10.1016/S0033-3182(95)71666-8. 7638314

[ref26] Hamilton, H. K., Boos, A. K., & Mathalon, D. H. (2020). Electroencephalography and event-related potential biomarkers in individuals at clinical high risk for psychosis. Biological Psychiatry, 88(4), 294–303. doi:10.1016/j.biopsych.2020.04.002. 32507388 PMC8300573

[ref27] Hamilton, H. K., Roach, B. J., Bachman, P. M., Belger, A., Carrion, R. E., Duncan, E., … Mathalon, D. H. (2019). Association between P300 responses to auditory oddball stimuli and clinical outcomes in the psychosis risk syndrome. JAMA Psychiatry, 76(11), 1187–1197. doi:10.1001/jamapsychiatry.2019.2135. 31389974 PMC6686970

[ref28] Higgins, A., Lewandowski, K. E., Liukasemsarn, S., & Hall, M. H. (2021). Longitudinal relationships between mismatch negativity, cognitive performance, and real-world functioning in early psychosis. Schizophrenia Research, 228, 385–393. doi:10.1016/j.schres.2021.01.009. 33549980 PMC7987838

[ref29] Ho, N. F., Holt, D. J., Cheung, M., Iglesias, J. E., Goh, A., Wang, M., … Zhou, J. (2017). Progressive decline in hippocampal CA1 volume in individuals at ultra-high-risk for psychosis who do not remit: Findings from the longitudinal youth at risk study. Neuropsychopharmacology, 42(6), 1361–1370. doi:10.1038/npp.2017.5. 28079061 PMC5437892

[ref30] Howes, O. D., McCutcheon, R., Agid, O., de Bartolomeis, A., van Beveren, N. J., Birnbaum, M. L., … Correll, C. U. (2017). Treatment-resistant schizophrenia: Treatment response and resistance in psychosis (TRRIP) working group consensus guidelines on diagnosis and terminology. American Journal of Psychiatry, 174(3), 216–229. doi:10.1176/appi.ajp.2016.16050503. 27919182 PMC6231547

[ref31] Javitt, D. C., & Freedman, R. (2015). Sensory processing dysfunction in the personal experience and neuronal machinery of schizophrenia. American Journal of Psychiatry, 172(1), 17–31. doi:10.1176/appi.ajp.2014.13121691. 25553496 PMC4501403

[ref32] Javitt, D. C., Steinschneider, M., Schroeder, C. E., & Arezzo, J. C. (1996). Role of cortical N-methyl-D-aspartate receptors in auditory sensory memory and mismatch negativity generation: Implications for schizophrenia. Proceedings of the National Academy of Sciences of the United States of America, 93(21), 11962–11967. doi:10.1073/pnas.93.21.11962. 8876245 PMC38166

[ref33] Jung, M. H., Jang, J. H., Kang, D. H., Choi, J. S., Shin, N. Y., Kim, H. S., … Kwon, J. S. (2010). The reliability and validity of the Korean version of the structured interview for prodromal syndrome. Psychiatry Investigation, 7(4), 257–263. doi:10.4306/pi.2010.7.4.257. 21253409 PMC3022312

[ref34] Kim, M., Cho, K. I., Yoon, Y. B., Lee, T. Y., & Kwon, J. S. (2017). Aberrant temporal behavior of mismatch negativity generators in schizophrenia patients and subjects at clinical high risk for psychosis. Clinical Neurophysiology, 128(2), 331–339. doi:10.1016/j.clinph.2016.11.027. 28056388

[ref35] Kim, M., Lee, T. H., Yoon, Y. B., Lee, T. Y., & Kwon, J. S. (2018). Predicting remission in subjects at clinical high risk for psychosis using mismatch negativity. Schizophrenia Bulletin, 44(3), 575–583. doi:10.1093/schbul/sbx102. 29036493 PMC5890455

[ref36] Kim, M., Lee, T. Y., Lee, S., Kim, S. N., & Kwon, J. S. (2015). Auditory P300 as a predictor of short-term prognosis in subjects at clinical high risk for psychosis. Schizophrenia Research, 165(2–3), 138–144. doi:10.1016/j.schres.2015.04.033. 25956629

[ref37] Koutsouleris, N., Riecher-Rossler, A., Meisenzahl, E. M., Smieskova, R., Studerus, E., Kambeitz-Ilankovic, L., … Borgwardt, S. (2015). Detecting the psychosis prodrome across high-risk populations using neuroanatomical biomarkers. Schizophrenia Bulletin, 41(2), 471–482. doi:10.1093/schbul/sbu078. 24914177 PMC4332937

[ref38] Koutsouleris, N., Upthegrove, R., & Wood, S. J. (2019). Importance of variable selection in multimodal prediction models in patients at clinical high risk for psychosis and recent onset depression-reply. JAMA Psychiatry, 76(3), 339–340. doi:10.1001/jamapsychiatry.2018.4237. 30725089

[ref39] Kwon, J. S., Byun, M. S., Lee, T. Y., & An, S. K. (2012). Early intervention in psychosis: Insights from Korea. Asian Journal of Psychiatry, 5(1), 98–105. doi:10.1016/j.ajp.2012.02.007. 26878954

[ref40] Lally, J., Ajnakina, O., Di Forti, M., Trotta, A., Demjaha, A., Kolliakou, A., … Murray, R. M. (2016). Two distinct patterns of treatment resistance: Clinical predictors of treatment resistance in first-episode schizophrenia spectrum psychoses. Psychological Medicine, 46(15), 3231–3240. doi:10.1017/S0033291716002014. 27605254

[ref41] Lappin, J. M., Morgan, C., Chalavi, S., Morgan, K. D., Reinders, A. A., Fearon, P., … Dazzan, P. (2014). Bilateral hippocampal increase following first-episode psychosis is associated with good clinical, functional and cognitive outcomes. Psychological Medicine, 44(6), 1279–1291. doi:10.1017/S0033291713001712 23866084

[ref42] Lho, S. K., Kim, M., Lee, T. H., Kwak, Y. B., & Kwon, J. S. (2019). Predicting prognosis in patients with first-episode psychosis using auditory P300: A 1-year follow-up study. Clinical Neurophysiology, 130(1), 46–54. doi:10.1016/j.clinph.2018.10.011. 30476710

[ref43] Lho, S. K., Kim, M., Park, J., Hwang, W. J., Moon, S. Y., Oh, S., & Kwon, J. S. (2020). Progressive impairment of mismatch negativity is reflective of underlying pathophysiological changes in patients with first-episode psychosis. Frontiers in Psychiatry, 11, 587. doi:10.3389/fpsyt.2020.00587. 32625126 PMC7314980

[ref44] Lieberman, J. A. (1993). Prediction of outcome in first-episode schizophrenia. The Journal of Clinical Psychiatry, 54(Suppl), 13–17. 8097192

[ref45] Lieberman, J. A., Perkins, D., Belger, A., Chakos, M., Jarskog, F., Boteva, K., & Gilmore, J. (2001). The early stages of schizophrenia: Speculations on pathogenesis, pathophysiology, and therapeutic approaches. Biological Psychiatry, 50(11), 884–897. doi:10.1016/s0006-3223(01)01303-8. 11743943

[ref46] Lin, A., Wood, S. J., Nelson, B., Beavan, A., McGorry, P., & Yung, A. R. (2015). Outcomes of nontransitioned cases in a sample at ultra-high risk for psychosis. American Journal of Psychiatry, 172(3), 249–258. doi:10.1176/appi.ajp.2014.13030418. 25727537

[ref47] Marshall, M., Lewis, S., Lockwood, A., Drake, R., Jones, P., & Croudace, T. (2005). Association between duration of untreated psychosis and outcome in cohorts of first-episode patients: A systematic review. Archives of General Psychiatry, 62(9), 975–983. doi:10.1001/archpsyc.62.9.975. 16143729

[ref48] Mi, L., Wang, L., Li, X., She, S., Li, H., Huang, H., … Zheng, Y. (2021). Reduction of phonetic mismatch negativity may depict illness course and predict functional outcomes in schizophrenia. Journal of Psychiatric Research, 137, 290–297. doi:10.1016/j.jpsychires.2021.02.065. 33735719

[ref49] Miller, T. J., McGlashan, T. H., Rosen, J. L., Cadenhead, K., Cannon, T., Ventura, J., … Woods, S. W. (2003). Prodromal assessment with the structured interview for prodromal syndromes and the scale of prodromal symptoms: Predictive validity, interrater reliability, and training to reliability. Schizophrenia Bulletin, 29(4), 703–715. doi:10.1093/oxfordjournals.schbul.a007040. 14989408

[ref50] Miller, T. J., McGlashan, T. H., Rosen, J. L., Somjee, L., Markovich, P. J., Stein, K., & Woods, S. W. (2002). Prospective diagnosis of the initial prodrome for schizophrenia based on the structured interview for prodromal syndromes: Preliminary evidence of interrater reliability and predictive validity. American Journal of Psychiatry, 159(5), 863–865. doi:10.1176/appi.ajp.159.5.863. 11986145

[ref51] Mouchlianitis, E., Bloomfield, M. A., Law, V., Beck, K., Selvaraj, S., Rasquinha, N., … Howes, O. D. (2016). Treatment-resistant schizophrenia patients show elevated anterior cingulate cortex glutamate compared to treatment-responsive. Schizophrenia Bulletin, 42(3), 744–752. doi:10.1093/schbul/sbv151. 26683625 PMC4838083

[ref52] Naatanen, R., & Escera, C. (2000). Mismatch negativity: Clinical and other applications. Audiology and Neurotology, 5(3–4), 105–110. doi:10.1159/000013874. 10859407

[ref53] Nagai, T., Tada, M., Kirihara, K., Yahata, N., Hashimoto, R., Araki, T., & Kasai, K. (2013). Auditory mismatch negativity and P3a in response to duration and frequency changes in the early stages of psychosis. Schizophrenia Research, 150(2–3), 547–554. doi:10.1016/j.schres.2013.08.005. 24012461

[ref54] Oh, S., Kim, M., Kim, T., Lee, T. Y., & Kwon, J. S. (2020). Resting-state functional connectivity of the striatum predicts improvement in negative symptoms and general functioning in patients with first-episode psychosis: A 1-year naturalistic follow-up study. Australian & New Zealand Journal of Psychiatry, 54(5), 509–518. doi:10.1177/0004867419885452. 31702384

[ref55] Oribe, N., Hirano, Y., Del Re, E., Mesholam-Gately, R. I., Woodberry, K. A., Ueno, T., … Niznikiewicz, M. A. (2020). Longitudinal evaluation of visual P300 amplitude in clinical high-risk subjects: An event-related potential study. Psychiatry and Clinical Neurosciences, 74(10), 527–534. doi:10.1111/pcn.13083. 32519778 PMC12832085

[ref56] Palaniyappan, L., Marques, T. R., Taylor, H., Handley, R., Mondelli, V., Bonaccorso, S., … Dazzan, P. (2013). Cortical folding defects as markers of poor treatment response in first-episode psychosis. JAMA Psychiatry, 70(10), 1031–1040. doi:10.1001/jamapsychiatry.2013.203. 23945954 PMC7617342

[ref57] Park, J., Lho, S. K., Hwang, W. J., Moon, S. Y., Oh, S., Kim, M., … Kwon, J. S. (2021). Impaired error-related processing in patients with first-episode psychosis and subjects at clinical high risk for psychosis: An event-related potential study. Psychiatry and Clinical Neuroscience. *75*, 219–226. doi:10.1111/pcn.13219. 33864656

[ref58] Perez, V. B., Woods, S. W., Roach, B. J., Ford, J. M., McGlashan, T. H., Srihari, V. H., & Mathalon, D. H. (2014). Automatic auditory processing deficits in schizophrenia and clinical high-risk patients: Forecasting psychosis risk with mismatch negativity. Biological Psychiatry, 75(6), 459–469. doi:10.1016/j.biopsych.2013.07.038. 24050720 PMC4028131

[ref59] Perkins, D. O., Gu, H., Boteva, K., & Lieberman, J. A. (2005). Relationship between duration of untreated psychosis and outcome in first-episode schizophrenia: A critical review and meta-analysis. American Journal of Psychiatry, 162(10), 1785–1804. doi:10.1176/appi.ajp.162.10.1785. 16199825

[ref60] Rapado-Castro, M., Dodd, S., Bush, A. I., Malhi, G. S., Skvarc, D. R., On, Z. X., … Dean, O. M. (2017). Cognitive effects of adjunctive N-acetyl cysteine in psychosis. Psychological Medicine, 47(5), 866–876. doi:10.1017/S0033291716002932. 27894373

[ref61] Renaldi, R., Kim, M., Lee, T. H., Kwak, Y. B., Tanra, A. J., & Kwon, J. S. (2019). Predicting symptomatic and functional improvements over 1 year in patients with first-episode psychosis using resting-state electroencephalography. Psychiatry Investigation, 16(9), 695–703. doi:10.30773/pi.2019.06.20.1. 31429218 PMC6761798

[ref62] Reniers, R. L., Lin, A., Yung, A. R., Koutsouleris, N., Nelson, B., Cropley, V. L., … Wood, S. J. (2017). Neuroanatomical predictors of functional outcome in individuals at ultra-high risk for psychosis. Schizophrenia Bulletin, 43(2), 449–458. doi:10.1093/schbul/sbw086. 27369472 PMC5605267

[ref63] Sarpal, D. K., Argyelan, M., Robinson, D. G., Szeszko, P. R., Karlsgodt, K. H., John, M., … Malhotra, A. K. (2016). Baseline striatal functional connectivity as a predictor of response to antipsychotic drug treatment. American Journal of Psychiatry, 173(1), 69–77. doi:10.1176/appi.ajp.2015.14121571. 26315980 PMC4845897

[ref64] Schlosser, D. A., Jacobson, S., Chen, Q., Sugar, C. A., Niendam, T. A., Li, G., … Cannon, T. D. (2012). Recovery from an at-risk state: Clinical and functional outcomes of putatively prodromal youth who do not develop psychosis. Schizophrenia Bulletin, 38(6), 1225–1233. doi:10.1093/schbul/sbr098. 21825282 PMC3494042

[ref65] Schmidt, A., Cappucciati, M., Radua, J., Rutigliano, G., Rocchetti, M., Dell'Osso, L., … Fusar-Poli, P. (2017). Improving prognostic accuracy in subjects at clinical high risk for psychosis: Systematic review of predictive models and meta-analytical sequential testing simulation. Schizophrenia Bulletin, 43(2), 375–388. doi:10.1093/schbul/sbw098. 27535081 PMC5605272

[ref66] Semlitsch, H. V., Anderer, P., Schuster, P., & Presslich, O. (1986). A solution for reliable and valid reduction of ocular artifacts, applied to the P300 ERP. Psychophysiology, 23(6), 695–703. doi:10.1111/j.1469-8986.1986.tb00696.x. 3823345

[ref67] Simon, A. E., Borgwardt, S., Riecher-Rossler, A., Velthorst, E., de Haan, L., & Fusar-Poli, P. (2013). Moving beyond transition outcomes: Meta-analysis of remission rates in individuals at high clinical risk for psychosis. Psychiatry Research, 209(3), 266–272. doi:10.1016/j.psychres.2013.03.004. 23871169

[ref68] Swerdlow, N. R., Bhakta, S., Chou, H. H., Talledo, J. A., Balvaneda, B., & Light, G. A. (2016). Memantine effects on sensorimotor gating and mismatch negativity in patients with chronic psychosis. Neuropsychopharmacology, 41(2), 419–430. doi:10.1038/npp.2015.162. 26062785 PMC5130118

[ref69] Tateno, T., Higuchi, Y., Nakajima, S., Sasabayashi, D., Nakamura, M., Ueno, M., … Suzuki, M. (2021). Features of duration mismatch negativity around the onset of overt psychotic disorders: A longitudinal study. Cerebral Cortex, 31(5), 2416–2424. doi:10.1093/cercor/bhaa364. 33341873

[ref70] Uno, Y., & Coyle, J. T. (2019). Glutamate hypothesis in schizophrenia. Psychiatry and Clinical Neuroscience, 73(5), 204–215. doi:10.1111/pcn.12823. 30666759

[ref71] Yung, A. R., Yuen, H. P., McGorry, P. D., Phillips, L. J., Kelly, D., Dell'Olio, M., … Buckby, J. (2005). Mapping the onset of psychosis: The comprehensive assessment of at-risk mental states. Australian & New Zealand Journal of Psychiatry, 39(11–12), 964–971. doi:10.1080/j.1440-1614.2005.01714.x. 16343296

[ref72] Zheng, W., Zhang, Q. E., Cai, D. B., Yang, X. H., Qiu, Y., Ungvari, G. S., … Xiang, Y. T. (2018). N-acetylcysteine for major mental disorders: A systematic review and meta-analysis of randomized controlled trials. Acta Psychiatrica Scandinavica, 137(5), 391–400. doi:10.1111/acps.12862. 29457216

