# Supplementary material for: Forecasting prognostic trajectories with mismatch negativity in early psychosis
Source: Psychol Med. 2021 Sep 2;53(4):1489–99. doi: 10.1017/S0033291721003068 (PMC10009395; doi:10.1017/S0033291721003068)
Supplement: Supplementary file 1 [file S0033291721003068sup001.docx]

**Forecasting prognostic trajectories with mismatch negativity in early psychosis**

**Supplementary Material**

Minah Kim^1,2^, Taekwan Kim^3^, Wu Jeong Hwang^3^, Silvia Kyungjin Lho^1,2^, Sun-Young Moon^1,2^, Tae Young Lee^4,5^, Jun Soo Kwon^1,2,3,5,*^

^1^Department of Neuropsychiatry, Seoul National University Hospital, Seoul, Republic of Korea

^2^Department of Psychiatry, Seoul National University College of Medicine, Seoul, Republic of Korea

^3^Department of Brain and Cognitive Sciences, Seoul National University College of Natural Sciences, Seoul, Republic of Korea

^4^Department of Neuropsychiatry, Pusan National University Yangsan Hospital, Yangsan, Republic of Korea

^5^Institute of Human Behavioral Medicine, SNU-MRC, Seoul, Republic of Korea

***Corresponding Author**

Professor Jun Soo Kwon, M.D., Ph.D.

Department of Psychiatry, Seoul National University College of Medicine, 101 Daehak-no, Chongno-gu, Seoul, Republic of Korea, 03080

Tel.: +82 2 2072 2972

Fax: +82 2 747 9063

E-mail: kwonjs@snu.ac.kr


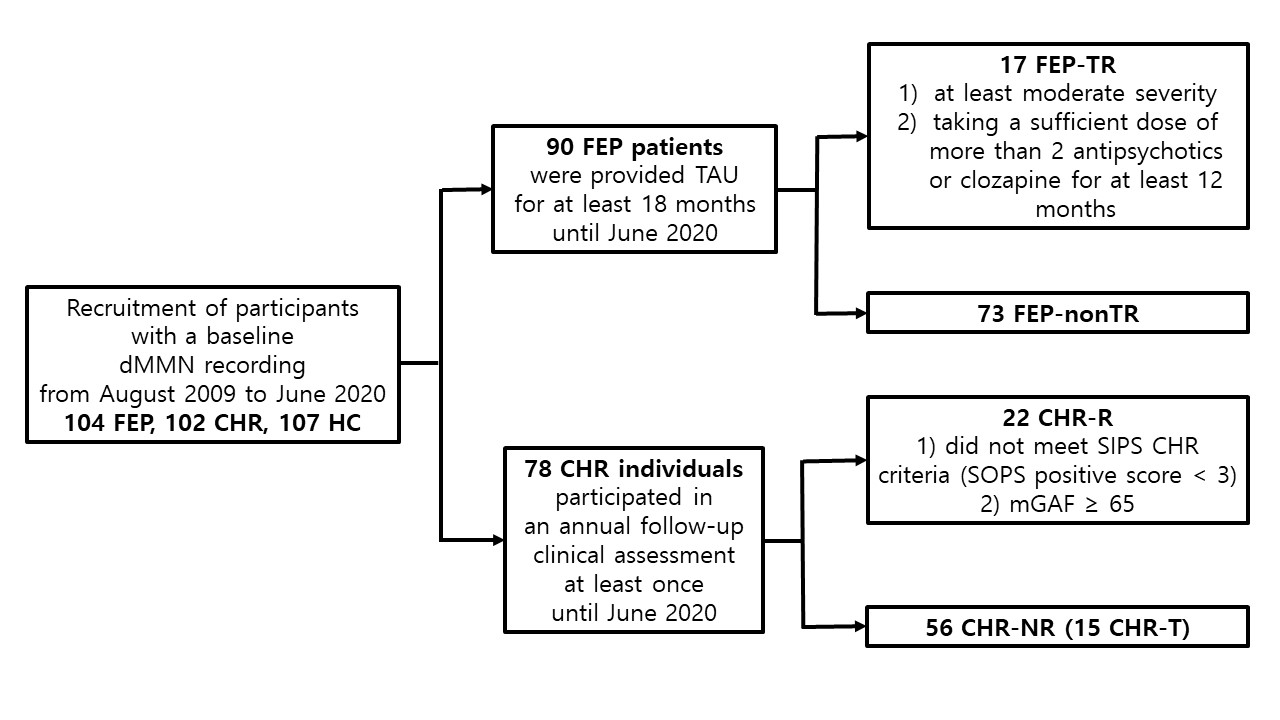


**Figure S1.** Recruitment and clinical outcome assessment procedures. dMMN, duration deviant mismatch negativity; FEP, first-episode psychosis; CHR, clinical high risk; HC, healthy control; TAU, treatment as usual; TR, treatment resistant; nonTR, not treatment resistant; R, remitters; SIPS, Structured Interview for Prodromal Syndromes; SOPS, Scale of Prodromal Symptoms; mGAF, modified Global Assessment of Functioning; NR, nonremitters; T, transitioned to psychotic disorder.


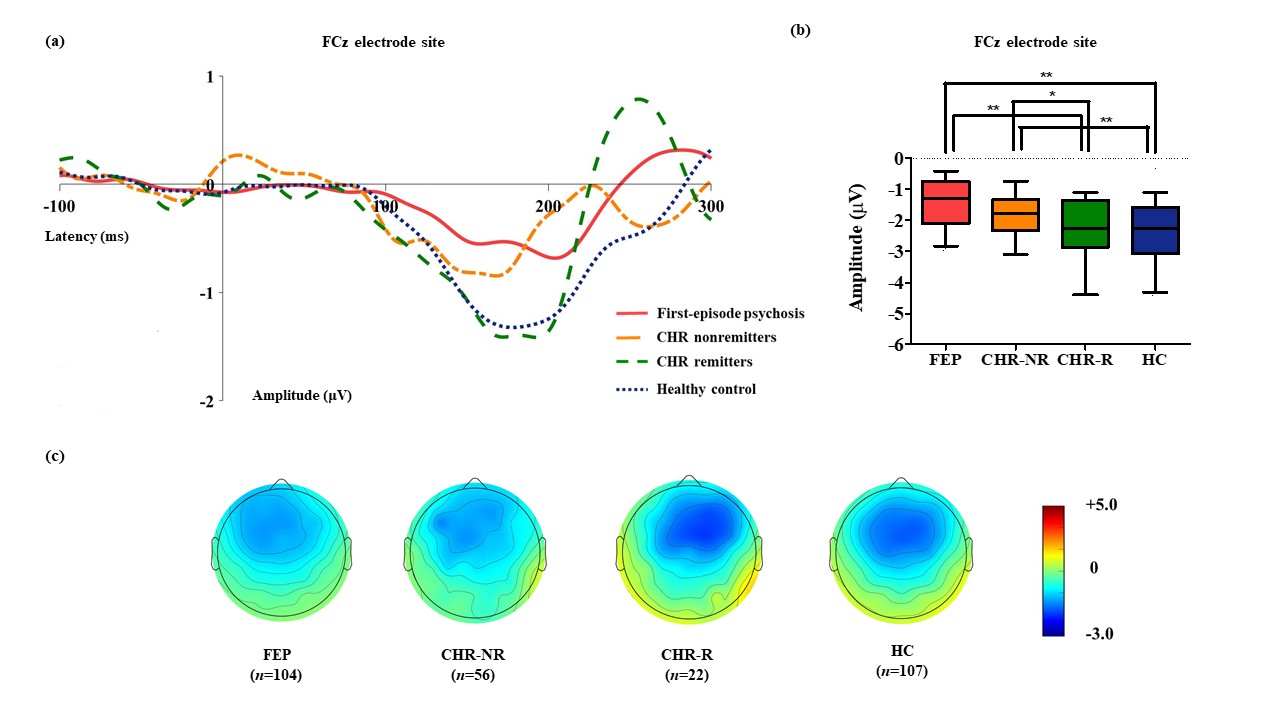


**Figure S2.** (a) Grand-averaged duration deviant mismatch negativity (dMMN) waveforms across first-episode psychosis (FEP) patients, nonremitted individuals at clinical high risk (CHR-NR) for psychosis, remitted CHR subjects (CHR-R), and healthy controls (HCs) at the FCz electrode site. (b) dMMN amplitude across the groups at the FCz electrode site. Analysis of covariance with age as a covariate showed that dMMN amplitudes were significantly different across FEP patients, CHR-NR subjects, CHR-R subjects, and HCs (F = 14.435, p < 0.001). A *post hoc* simple contrast test revealed that dMMN amplitudes were similarly impaired in FEP patients and CHR-NR subjects compared to CHR-R subjects and HCs (FEP vs CHR-NR, p = 0.164; FEP vs CHR-R, p = 0.004; CHR-R vs HC, p = 0.441). Horizontal lines in groups indicate means, and vertical lines indicate 95% confidence intervals. * indicates statistical significance at p < 0.05. ** indicates statistical significance at p < 0.005. (c) Two-dimensional topographic maps of dMMN in FEP patients, CHR-NR subjects, CHR-R subjects, and HCs. The colored bar with numbers indicates the dMMN amplitude (μV).


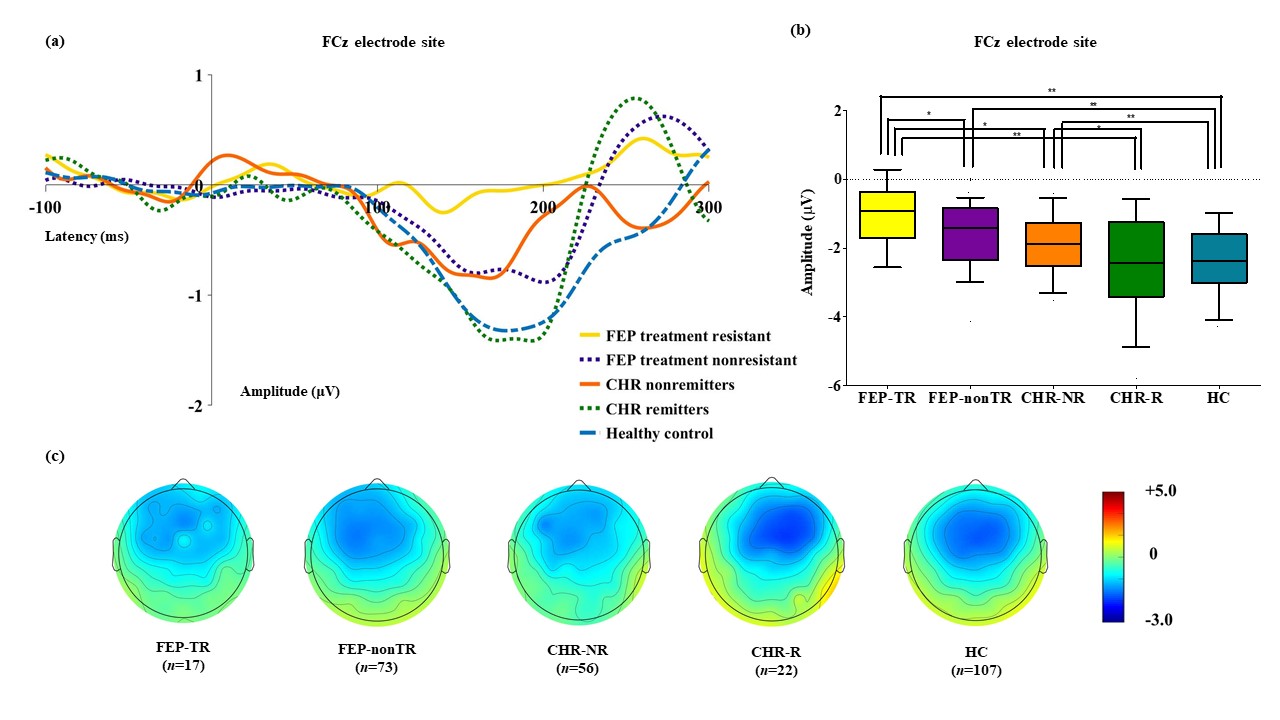


**Figure S3.** (a) Grand-averaged duration-deviant mismatch negativity (dMMN) waveforms across treatment-resistant first-episode psychosis (FEP-TR) patients, treatment-nonresistant FEP patients (FEP-nonTR), nonremitted individuals at clinical high risk (CHR-NR) for psychosis, remitted CHR subjects (CHR-R), and healthy controls (HCs) at the FCz electrode site. (b) dMMN amplitude across the groups at the FCz electrode site. Analysis of covariance with age as a covariate showed that dMMN amplitudes were significantly different across FEP-TR patients, FEP-nonTR patients, CHR-NR subjects, CHR-R subjects, and HCs (F = 10.169, p < 0.001). A *post hoc* simple contrast test revealed that dMMN amplitudes were smallest in FEP-TR patients (FEP-TR vs FEP-nonTR, p = 0.029; FEP-TR vs CHR-NR, p = 0.020; FEP-TR vs CHR-R, p = 0.001; FEP-TR vs HC, p < 0.001), intermediate in FEP-nonTR patients and CHR-NR subjects (FEP-nonTR vs CHR-NR, p = 0.718; FEP-nonTR vs CHR-R, p = 0.043; FEP-nonTR vs HC, p < 0.001), and largest in CHR-R subjects and HCs (CHR-R vs HC, p = 0.431). Horizontal lines in groups indicate means, and vertical lines indicate 95% confidence intervals. * indicates statistical significance at p < 0.05. ** indicates statistical significance at p < 0.005. (c) Two-dimensional topographic maps of dMMN in FEP-TR patients, FEP-nonTR patients, CHR-NR subjects, CHR-R subjects, and HCs. The colored bar with numbers indicates the dMMN amplitude (μV).

**Table S1.** Demographic and clinical characteristics of patients with first-episode psychosis (FEP) who were and were not treatment resistant (FEP-TR and FEP-nonTR).

|  | FEP-TR | FEP-nonTR | Statistical analysis^a^ | |
| --- | --- | --- | --- | --- |
| Characteristics | (n = 17) | (n = 73) | χ^2^ or Z | P |
| Sex (male/female) | 8/9 | 29/44 | 0.306 | 0.580 |
| Handedness (right/left) | 15/2 | 69/4 | 0.875 | 0.349 |
| Age (years) | 23.2 ± 4.5 | 23.6 ± 5.2 | -0.201 | 0.840 |
| IQ | 96.8 ± 18.3 | 101.6 ± 13.5 | -1.516 | 0.129 |
| Education (years) | 14.2 ± 3.9 | 13.9 ± 2.3 | -0.404 | 0.686 |
| DOI (months) | 10.6 ± 5.9 | 6.9 ± 5.1 | -2.377 | 0.017* |
| DUP (months) | 5.7 ± 5.4 | 4.0 ± 4.2 | -0.745 | 0.457 |
| *Baseline characteristics* |  |  |  |  |
| PANSS at baseline |  |  |  |  |
| Positive symptoms | 16.9 ± 5.0 | 14.5 ± 5.4 | -1.751 | 0.080 |
| Negative symptoms | 16.5 ± 6.3 | 16.1 ± 5.9 | -0.341 | 0.733 |
| General symptoms | 29.9 ± 9.2 | 31.7 ± 8.5 | -0.671 | 0.502 |
| mGAF at baseline | 46.8 ± 16.0 | 51.7 ± 12.7 | -1.181 | 0.237 |
| Medication use at baseline^b^ |  |  |  |  |
| Antipsychotics | 17 (100.0) | 65 (89.0) | 2.045 | 0.153 |
| Antidepressants | 3 (17.6) | 8 (11.0) | 0.575 | 0.448 |
| Mood stabilizers | 2 (11.8) | 7 (9.6) | 1.540 | 0.215 |
| Anxiolytics | 11 (64.7) | 41 (56.2) | 0.412 | 0.521 |
| Antipsychotics dose at baseline^c^ | 20.0 ± 12.1 | 11.4 ± 8.4 | -2.884 | 0.004** |
| Diagnosis at baseline^d^ |  |  |  |  |
| Schizophrenia   /schizophreniform disorder | 16 (94.1) | 66 (90.4) | 0.234 | 0.629 |
| Schizoaffective disorder | 1 (5.9) | 7 (9.6) |  |  |
| *Follow-up characteristics* |  |  |  |  |
| Follow-up duration (months) | 39.4 ± 32.7 | 46.1 ± 34.2 | -0.734 | 0.465 |
| Medication use at last follow-up^e^ |  |  |  |  |
| Clozapine | 10 (58.8) | 0 (0.0) | 48.309 | <0.001** |
| Multiple antipsychotics | 12 (70.6) | 21 (28.8) | 10.385 | 0.001** |
| Antipsychotics | 17 (100.0) | 70 (95.9) | 0.723 | 0.395 |
| Antipsychotics dose at last follow-up^f^ | 24.0 ± 11.1 | 11.6 ± 8.6 | -4.095 | <0.001** |

Abbreviations: IQ, intelligence quotient; DOI, duration of illness; DUP, duration of untreated psychosis; PANSS, Positive and Negative Syndrome Scale; mGAF, Modified Global Assessment of Functioning.

^a^ Mann-Whitney U test, χ2 analysis or Fisher's exact test for categorical data.

^b^ Number (percentage) of FEP patients who were prescribed each medication at baseline.

^c^ Mean daily olanzapine equivalent dose prescribed at baseline.

^d^ Number (percentage) of FEP patients who met each diagnosis at baseline when assessed with the Structured Clinical Interview for the Diagnostic and Statistical Manual of Mental Disorders, Fourth Edition, Axis I Disorders (SCID-I).

^e^ Number (percentage) of FEP patients who were prescribed each medication at last follow-up.

^f^ Mean olanzapine equivalent dose prescribed at the last follow-up.

Data are presented as the mean ± standard deviation.

*, statistical significance at p < 0.05.

**, statistical significance at p < 0.005.

**Table S2.** Demographic and clinical characteristics of subjects at clinical high risk (CHR) for psychosis who did and did not remit and those who did and did not transition to psychotic disorder (CHR-T and CHR-NT).

|  | CHR remitters | CHR nonremitters | Statistical analysis^a^ | | CHR-T | CHR-NT | Statistical analysis^b^ | |
| --- | --- | --- | --- | --- | --- | --- | --- | --- |
| Characteristics | (n = 22) | (n = 56) | χ^2^ or T | P | (n = 15) | (n = 63) | χ^2^ or Z | P |
| Sex (male/female) | 16/6 | 40/16 | 0.013 | 0.909 | 9/6 | 47/16 | 1.276 | 0.259 |
| Handedness (right/left) | 22/0 | 52/4 | 1.656 | 0.198 | 13/2 | 61/2 | 2.570 | 0.109 |
| Age (years) | 19.9 ± 3.1 | 20.5 ± 3.8 | -0.643 | 0.522 | 21.2 ± 4.6 | 20.1 ± 3.3 | -0.564 | 0.573 |
| IQ | 105.8 ± 14.6 | 104.3 ± 12.0 | 0.474 | 0.637 | 105.5 ± 11.2 | 104.5 ± 13.1 | -0.152 | 0.879 |
| Education (years) | 12.5 ± 1.4 | 12.6 ± 1.8 | -0.244 | 0.808 | 12.7 ± 2.0 | 12.5 ± 1.7 | -0.085 | 0.932 |
| DUPP (months) | 19.5 ± 22.9 | 17.4 ± 16.4 | 0.455 | 0.651 | 17.5 ± 16.4 | 18.1 ± 18.9 | -0.527 | 0.598 |
| Prodromal syndromes^c^ |  |  |  |  |  |  |  |  |
| APS | 18 (81.8) | 46 (82.1) | 0.001 | 0.973 | 12 (80.0) | 52 (82.5) | 0.053 | 0.818 |
| BIPS | 1 (4.5) | 1 (1.8) | 0.482 | 0.488 | 1 (6.7) | 1 (1.6) | 1.251 | 0.263 |
| GRD | 3 (13.6) | 15 (26.8) | 1.538 | 0.215 | 4 (26.7) | 14 (22.2) | 0.135 | 0.713 |
| *Baseline characteristics* |  |  |  |  |  |  |  |  |
| SOPS at baseline |  |  |  |  |  |  |  |  |
| Positive symptoms | 9.3 ± 3.6 | 9.8 ± 3.8 | -0.476 | 0.636 | 11.5 ± 3.7 | 9.2 ± 3.6 | -2.018 | 0.044* |
| Negative symptoms | 13.3 ± 7.1 | 14.3 ± 6.5 | -0.612 | 0.542 | 13.7 ± 6.2 | 14.1 ± 6.8 | -0.318 | 0.751 |
| Disorganization | 4.3 ± 2.6 | 4.6 ± 3.1 | -0.390 | 0.698 | 5.0 ± 2.2 | 4.4 ± 3.1 | -1.164 | 0.244 |
| General symptoms | 7.0 ± 3.8 | 7.3 ± 4.1 | -0.281 | 0.779 | 7.5 ± 4.7 | 7.1 ± 3.9 | -0.419 | 0.675 |
| mGAF at baseline | 50.3 ± 9.5 | 52.0 ± 9.0 | -0.769 | 0.444 | 52.2 ± 9.6 | 51.4 ± 9.0 | -0.337 | 0.736 |
| Medication use at baseline^d^ |  |  |  |  |  |  |  |  |
| Antipsychotics | 3 (13.6) | 7 (12.5) | 0.018 | 0.893 | 3 (20.0) | 7 (11.1) | 0.856 | 0.355 |
| Antidepressants | 4 (18.2) | 17 (30.4) | 1.190 | 0.275 | 4 (26.7) | 17 (30.0) | 0.001 | 0.980 |
| Mood stabilizers | 1 (4.5) | 1 (1.8) | 0.482 | 0.488 | 1 (6.7) | 1 (1.6) | 1.251 | 0.263 |
| Anxiolytics | 5 (22.7) | 17 (30.4) | 0.454 | 0.500 | 6 (40.0) | 16 (15.9) | 1.276 | 0.259 |
| *Follow-up characteristics* |  |  |  |  |  |  |  |  |
| Follow-up duration (months) | 46.0 ± 36.5 | 30.1 ± 27.9 | 1.843 | 0.075 | 41.7 ± 37.4 | 32.9 ± 29.5 | -0.767 | 0.443 |
| SOPS positive symptoms | 2.0 ± 2.0 | 9.1 ± 6.3 | -7.202 | <0.001** | - | - | - | - |
| mGAF | 72.8 ± 6.2 | 56.9 ± 11.6 | 7.627 | <0.001** | - | - | - | - |
| Medication use during follow-up^e^ |  |  |  |  |  |  |  |  |
| Antipsychotics | 14 (63.6) | 48 (85.7) | 3.032 | 0.082 | - | - | - | - |
| Antidepressants | 15 (68.2) | 29 (51.8) | 2.180 | 0.140 | - | - | - | - |
| Mood stabilizers | 11 (50.0) | 30 (53.6) | 0.029 | 0.866 | - | - | - | - |
| Anxiolytics | 8 (36.4) | 28 (50.0) | 1.001 | 0.317 | - | - | - | - |
| Antipsychotic dose^f^ | 2.1 ± 2.2 | 5.3 ± 4.8 | -3.910 | <0.001** | - | - | - | - |

Abbreviations: IQ, intelligence quotient; DUPP, duration of untreated prodromal psychosis; APS, attenuated positive symptom; BIPS, brief intermittent psychotic symptom; GRD, genetic risk and deterioration; SOPS, Scale of Prodromal Symptoms; mGAF, modified Global Assessment of Functioning.

^a^ Independent t-test or Welch's t-test if the variances were not equal; χ2 analysis or Fisher's exact test for categorical data.

^b^ Mann-Whitney U test; χ2 analysis or Fisher's exact test for categorical data.

^c^ Prodromal syndromes for APS, BIPS, and GRD are not mutually exclusive according to the SIPS criteria.

^d^ Number (percentage) of CHR subjects who were prescribed each medication at baseline.

^e^ Number (percentage) of CHR subjects who were prescribed each medication during the follow-up period.

^f^ Mean daily olanzapine equivalent dose prescribed during the follow-up period.

Data are presented as the mean ± standard deviation.

*, statistical significance at p < 0.05.

**, statistical significance at p < 0.005.

**Table S3.** Demographic and clinical characteristics of patients with first-episode psychosis (FEP) who were or were not assessed for treatment resistance (FEP-A and FEP-NA).

|  | FEP-A | FEP-NA | Statistical analysis^a^ | |
| --- | --- | --- | --- | --- |
| Characteristics | (n = 90) | (n = 14) | χ^2^ or Z | P |
| Sex (male/female) | 37/53 | 4/10 | 0.798 | 0.372 |
| Handedness (right/left) | 84/6 | 13/1 | 0.004 | 0.947 |
| Age (years) | 23.5 ± 5.0 | 22.6 ± 3.0 | -0.573 | 0.567 |
| IQ | 100.7 ± 14.5 | 97.3 ± 15.6 | -0.777 | 0.437 |
| Education (years) | 13.9 ± 2.7 | 14.4 ± 2.1 | -0.992 | 0.321 |
| DOI (months) | 7.6 ± 5.4 | 12.0 ± 9.7 | -1.417 | 0.156 |
| DUP (months) | 4.3 ± 4.5 | 6.5 ± 5.8 | -1.685 | 0.092 |
| PANSS |  |  |  |  |
| Positive symptoms | 14.9 ± 5.3 | 16.8 ± 6.4 | -0.926 | 0.355 |
| Negative symptoms | 16.1 ± 6.0 | 15.7 ± 6.8 | -0.386 | 0.699 |
| General symptoms | 31.4 ± 8.6 | 34.6 ± 11.7 | -0.724 | 0.469 |
| mGAF | 50.7 ± 13.4 | 50.8 ± 10.9 | -0.014 | 0.989 |
| Medication use^b^ |  |  |  |  |
| Antipsychotics | 82 (91.1) | 13 (92.9) | 0.047 | 0.829 |
| Antidepressants | 11 (12.2) | 1 (7.1) | 0.306 | 0.580 |
| Mood stabilizers | 5 (5.6) | 5 (35.7) | 12.680 | <0.001** |
| Anxiolytics | 52 (57.8) | 9 (64.3) | 0.212 | 0.646 |

Abbreviations: IQ, intelligence quotient; DOI, duration of illness; DUP, duration of untreated psychosis; PANSS, Positive and Negative Syndrome Scale; mGAF, modified Global Assessment of Functioning.

^a^ Mann-Whitney U test; χ2 analysis or Fisher's exact test for categorical data.

^b^ Number (percentage) of FEP patients who were prescribed each medication at baseline.

Data are presented as the mean ± standard deviation.

**, statistical significance at p < 0.005.

**Table S4.** Demographic and clinical characteristics of subjects at clinical high risk (CHR) for psychosis who did and did not participate in the follow-up assessment (CHR-F and CHR-NF).

|  | CHR-F | CHR-NF | Statistical analysis^a^ | |
| --- | --- | --- | --- | --- |
| Characteristics | (n = 78) | (n = 24) | χ^2^ or T | P |
| Sex (male/female) | 56/22 | 19/5 | 0.512 | 0.474 |
| Handedness (right/left) | 74/4 | 23/1 | 0.036 | 0.849 |
| Age (years) | 20.3 ± 3.6 | 20.0 ± 4.0 | 0.376 | 0.708 |
| IQ | 104.7 ± 12.7 | 108.8 ± 15.0 | -1.318 | 0.191 |
| Education (years) | 12.6 ± 1.7 | 12.3 ± 2.1 | 0.765 | 0.446 |
| DUPP (months) | 17.9 ± 18.3 | 17.3 ± 16.3 | 0.166 | 0.868 |
| SOPS |  |  |  |  |
| Positive symptoms | 9.6 ± 3.7 | 10.2 ± 3.2 | -0.667 | 0.506 |
| Negative symptoms | 14.0 ± 6.7 | 13.2 ± 6.8 | 0.541 | 0.589 |
| Disorganization | 4.5 ± 2.9 | 3.5 ± 2.9 | 1.445 | 0.152 |
| General symptoms | 7.2 ± 4.0 | 6.0 ± 3.4 | 1.377 | 0.171 |
| mGAF | 51.5 ± 9.0 | 58.9 ± 9.3 | -3.442 | 0.001** |
| Medication use^b^ |  |  |  |  |
| Antipsychotics | 10 (12.8) | 4 (16.7) | 0.229 | 0.632 |
| Antidepressants | 21 (26.9) | 4 (16.7) | 1.043 | 0.307 |
| Mood stabilizers | 2 (2.6) | 1 (4.2) | 0.165 | 0.684 |
| Anxiolytics | 22 (28.2) | 4 (16.7) | 1.287 | 0.257 |

Abbreviations: IQ, intelligence quotient; DUPP, duration of untreated prodromal psychosis; SOPS, Scale of Prodromal Symptoms; mGAF, modified Global Assessment of Functioning.

^a^ Independent t-test or Welch's t-test if the variances were not equal, χ2 analysis or Fisher's exact test for categorical data.

^b^ Number (percentage) of CHR subjects who were prescribed each medication at baseline.

Data are given as the mean ± standard deviation.

**, statistical significance at p < 0.005.

**Table S5.** Mismatch negativity (MMN) characteristics of subjects at clinical high risk (CHR) for psychosis who did and did not transition to psychotic disorder (CHR-T and CHR-NT).

|  | CHR-T | CHR-NT | Statistical analysis^a^ | |
| --- | --- | --- | --- | --- |
|  | (n = 15) | (n = 63) | Z | P |
| *Peak amplitudes (μV)* |  |  |  |  |
| FCz electrode site | -2.1 ± 0.8 | -1.9 ± 1.0 | -1.084 | 0.278 |
| *Peak latencies (ms)* |  |  |  |  |
| FCz electrode site | 184.6 ± 33.8 | 191.1 ± 29.8 | -0.488 | 0.625 |
| Number of epochs for  deviant stimuli | 184.9 ± 33.6 | 194.6 ± 23.6 | -0.761 | 0.447 |

^a^ Mann-Whitney U test.

Data are presented as the mean ± standard deviation.

**Table S6.** Mismatch negativity (MMN) characteristics of patients with first-episode psychosis (FEP) who were and were not assessed for treatment resistance (FEP-A and FEP-NA).

|  | FEP-A | FEP-NA | Statistical analysis^a^ | |
| --- | --- | --- | --- | --- |
|  | (n = 90) | (n = 14) | Z | P |
| *Peak amplitudes (μV)* |  |  |  |  |
| FCz electrode site | -1.6 ± 1.1 | -1.1 ± 0.6 | -1.514 | 0.130 |
| *Peak latencies (ms)* |  |  |  |  |
| FCz electrode site | 178.4 ± 30.2 | 227.4 ± 21.6 | -4.801 | <0.001** |
| Number of epochs for  deviant stimuli | 192.7 ± 25.9 | 192.3 ± 24.6 | -0.500 | 0.617 |

^a^ Mann-Whitney U test.

Data are presented as the mean ± standard deviation.

**, statistical significance at p < 0.005.

**Table S7.** Mismatch negativity (MMN) characteristics of subjects at clinical high risk (CHR) for psychosis who did and did not participate in the follow-up assessment (CHR-F and CHR-NF).

|  | CHR-F | CHR-NF | Statistical analysis^a^ | |
| --- | --- | --- | --- | --- |
|  | (n = 78) | (n = 24) | T | P |
| *Peak amplitudes (μV)* |  |  |  |  |
| FCz electrode site | -2.0 ± 1.0 | -2.2 ± 1.1 | 1.065 | 0.290 |
| *Peak latencies (ms)* |  |  |  |  |
| FCz electrode site | 189.9 ± 30.5 | 199.5 ± 27.5 | -1.386 | 0.169 |
| Number of epochs for  deviant stimuli | 192.7 ± 25.9 | 192.3 ± 24.6 | 0.060 | 0.952 |

^a^ Independent t-test or Welch's t-test if the variances were not equal.

Data are presented as the mean ± standard deviation.
